# Almost 30 years of effort to bind clinical practice and science in the field of psychiatry in Europe

**DOI:** 10.1192/j.eurpsy.2022.1596

**Published:** 2022-09-01

**Authors:** M.B. Demirtas, E.C. Esen

**Affiliations:** 1 University of Health Sciences, Gulhane Training and Research Hospital, Psychiatry Department, Ankara, Turkey; 2 Balikesir Universitesi Egitim ve Arastirma Hastanesi 1.Kat Psikiyatri AD., Altieylul/ Balikesir, Balikesir, Turkey

**Keywords:** brain drain, utaut, e-mental health, telepsychiatry

## Abstract

**Introduction:**

European Federation of Psychiatric Trainees (EFPT) is a platform for psychiatric trainees from not only Europe but also various other countries. EFPT exclusively works on binding clinical practice and science for better mental health care. Research Working Group (RWG) of EFPT works on sharing knowledge with peers by brainstorming, collaborating and coordinating projects, organizing journal clubs and workshops.

**Objectives:**

We will focus on tele-psychiatry also known as e-mental health, a subdivision of telemedicine, provides diagnostic interview, evaluation, therapies, psycho-education and treatment. We plan The Brain-Drain follow-up study, investigates immigration of psychiatric trainees. Also educational activities have planned.

**Methods:**

With a questionnaire on the topic of psychiatry residents’ acceptance of tele-psychiatry using The Unified Theory of Acceptance and Use of Technology (UTAUT), we will hold the first multi-national survey among psychiatry residents. The Brain-Drain study was conducted by the EFPT-RWG in the past had a promising outcome. We are currently working on the follow-up of the study. We are starting to hold events. For instance we will commence the monthly journal club. Apart from giving a platform for scientific debate, journal club will also provide a chance have a elaborate discussion with author. We will organize a workshop on how to write a case-study with Neuro-Psycho-Pharmacology Working Group of EFPT.

**Results:**

We assume diverse attitudes overlapping different telepsychiatry exposure and regualtions, comprehensive data on immigration of trainees and sharing knowledge on practice and research.

**Conclusions:**

Hopefully, we will have clearer understanding of changes in working environment of trainees either with new technologies or in different countries.

**Disclosure:**

No significant relationships.

